# Supplementary material for: Efficient Genome Editing Using the T2A-Coupled Co-Expression of Two ZFN Monomers
Source: Int J Mol Sci. 2025 Aug 6;26(15):7602. doi: 10.3390/ijms26157602 (PMC12347167; doi:10.3390/ijms26157602)
Supplement: Supplementary file 1 [file ijms-26-07602-s001.zip › Katayama et al-Supplementary Table (IJMS).pdf]

Table S1. Primer sequences

Primer	Sequence (5' to 3')	Applications
inverse rev	gaagatgatctcatcgttgttgaacttatc	Construction
inverse fw	TAAGGGCCCTTCGAAGGTAAGCCTATCCCT	Construction
insert P2A fw	gatgagatcatcttcGGAAGCGGAgctactaacttcagcctgctgaagcaggctggagacgtggaggagaaccctggacctATGGGACCTAAGAAAAAGAGGAAGGTGGCG	Construction
insert T2A fw	gatgagatcatcttcGGAAGCGGAgagggcagaggaagtcttctaacatgcggtgacgtggaggagaatcccggccctATGGGACCTAAGAAAAAGAGGAAGGTGGCG	Construction
insert rev	TTCGAAGGGCCCTTAgaagatgatctcatcgttgttgaacttatc	Construction

Table S2. Primer sequences

Primer	Sequence (5' to 3')	Applications
AAVS-1 Fw	cgatgtccggagaggatggc	T7EI assay
AAVS-1 Rv	gatggtaaggaggactgcatgg	T7EI assay
2027ot1 fw	GAAGAGAATGAAGCGGCGGC	Off-target analysis
2027ot1 rv	ACTGACACCGAGACAGAGCA	Off-target analysis
2027ot2 fw	GAGGAGGCTCCCATCTGGCA	Off-target analysis
2027ot2 rv	TGTCTGAGGCAGGGGTAACC	Off-target analysis
9299v2ot1 fw	CCGCGCAGGATCCATTTTG	Off-target analysis
9299v2ot1 rv	CATCTTCCAGCACCTGCCAC	Off-target analysis
9299v2ot2 fw	GGGCTCCAAGCAGACTGAAGT	Off-target analysis
9299v2ot2 rv	CCCACCGGGAGAAGCTTATC	Off-target analysis
