# Supplementary material for: Efficient Genome Editing Using the T2A-Coupled Co-Expression of Two ZFN Monomers
Source: Int J Mol Sci. 2025 Aug 6;26(15):7602. doi: 10.3390/ijms26157602 (PMC12347167; doi:10.3390/ijms26157602)
Supplement: Supplementary file 1 [file ijms-26-07602-s001.zip › Katayama et al-Supplementary_Figure丂Production.pdf]

Figure S1 (Katayama et al.)

(a)

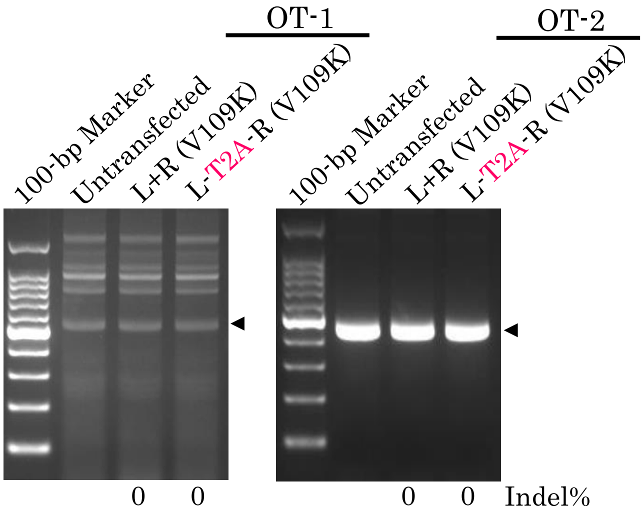

(b)

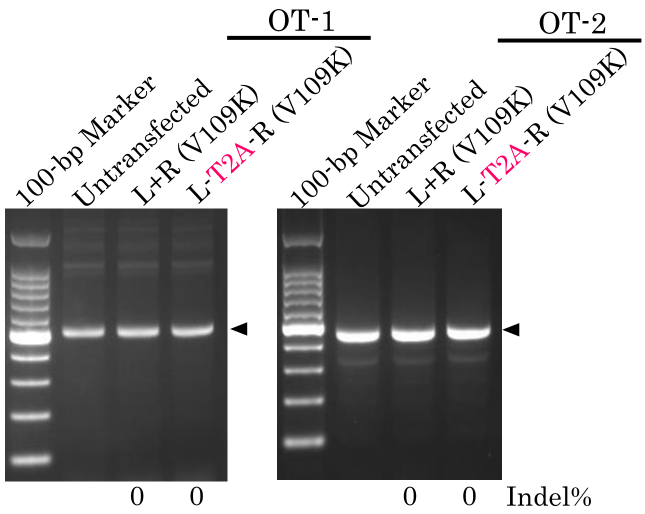

(c)

Target gene	Prototype	Name	Sequence (Top strand: 5' to 3', Bottom strand: 3' to 5')	Coordinate	Genomic region	Mutation	Identities (%)
Human AAVS-1	2027L+R	On-target	CAG CGC CCC CCG CCC GGC GTCTCC CGG GGC CAG GTC CAC CCT<br>GTC GCG GGG GGC GGG CCG CAGAGG GCC CCG GTC CAG GTG GGA	Chr19: 55117179-55117220	Intron	-	-
		OT1	CAG CGC CCC CCG CCC GGC CGCCGC TCG GAG CCA CCG CCC GCC<br>GTC GCG GGG GGC GGG CCG GCGGCG AGC CTC GGT GGC GGG CGG	Chr3: 46882282-46882323	Intergenic	Not detected	66.7
		OT2	TCT GGA GGA CGG GGA GGC AGCCCC AGG GGC CAG GTC CAC CCT<br>AGA CCT CCT GCC CCT CCG TCGGGG TCC CCG GTC CAG GTG GGA	Chr8: 143876342-143876383	Intron	Not detected	63.9
	9299L(v2) +R	On-target	CCC GTT GCC GTC TCG ATG CGCCCC GTC GTT CCT GGC CCT GGG<br>GGG CAA CGG CAG AGC TAG GCGGGG CAG CAA GGA CCG GGA CCC	Chr19: 55117107-55117149	Intron	-	-
		OT1	GCC GTT GCC GTC TAG ATC CAGCCG ATA AAG GGC TGG GCT GGC<br>CGG CAA CGG CAG ATC TAG GTCGGC TAT TTC CCG ACC CGA CCG	Chr7: 28956967-28957008	Exon	Not detected	61.1
		OT2	GCA TAG GAT TGC CAA GTA CCAGGA CTC GTT CCT GGC CCT GGA<br>CGT ATC CTA ACG GTT CAT GGTCCT GAG CAA GGA CCG GGA CCT	Chr12: 78280388-782803429	Intergenic	Not detected	55.6

Figure S1. Off-target analysis of ZF-ND1s. (**a, b**) T7E1 assay. A gel image of T7E1-treated PCR products amplified from the target sites. The arrowheads indicate the corresponding band for the target site. OT1 and 2 indicate the off-target candidate sites 1 and 2, respectively. The cells untransfected and transfected with L+R (V109K) or L-T2A-R (V109K) (**a**) were subjected to a T7E1 assay. The cells untransfected and transfected with L(v2)+R or L(v2)-T2A-R (**b**) were subjected to a T7E1 assay. (**c**) Summary of an off-target analysis of ZF-ND1s. The red and blue letters indicate left and right ZF-ND1 target sequences, respectively. Mismatches are underlined. Identities were calculated as follows: ((the number of match sequences) / 36) × 100.

Figure S2 (Katayama et al.)

(a)

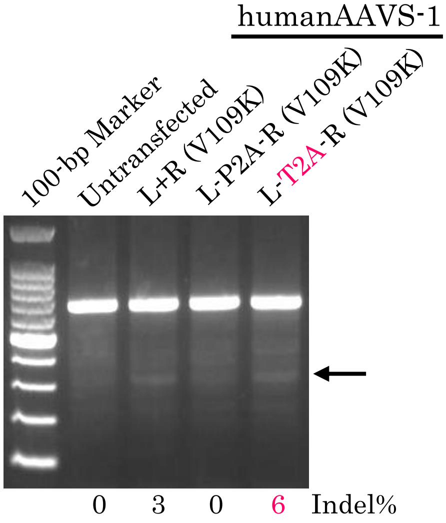

(b)

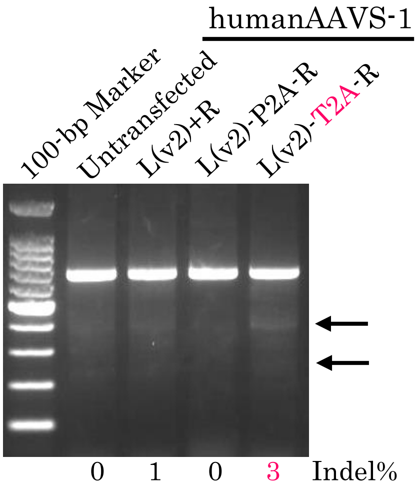

Figure S2. 2A-coupled co-expression of two ZF-ND1 monomers at a lower dose.
(**a, b**) The T7E1 assay. Gel images of T7E1-treated PCR products amplified from the target sites. Arrowheads indicate the cleaved DNA band. (**a**) L+R (V109K) indicates the separately expressed ZF-ND1 monomers. L-P2A-R (V109K) and L-T2A-R (V109K) indicate the P2A- and T2A-coupled ZF-ND1 monomers, respectively. The total amount of transfected plasmid DNA: L+R (V109K), 50 ng; L-P2A-R (V109K), 50 ng; L-T2A-R (V109K), 50 ng. (**b**) L(v2)+R indicates the separately expressed ZF-ND1 monomers. L(v2)-P2A-R and L(v2)-T2A-R indicate the P2A- and T2A-coupled ZF-ND1 monomers, respectively. The total amount of transfected plasmid DNA: L(v2)+R, 50 ng; L(v2)-P2A-R, 50 ng; L(v2)-T2A-R, 50 ng.

Figure S3 (Katayama et al.)

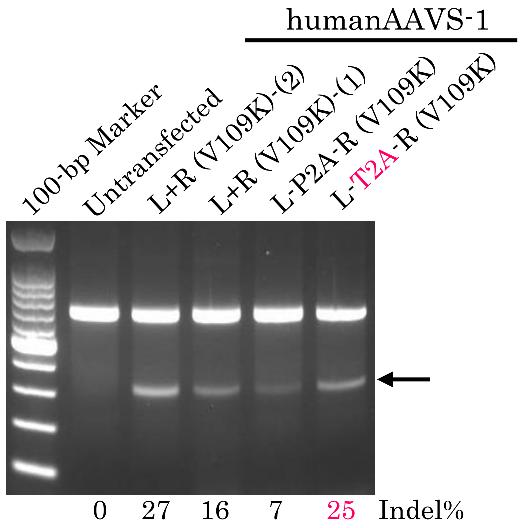

Figure S3. 2A-coupled co-expression of two ZF-ND1 monomers in Jurkat cells.
The T7E1 assay. Gel images of T7E1-treated PCR products amplified from the target sites. Arrowheads indicate the cleaved DNA band. L+R (V109K) indicates the separately expressed ZF-ND1 monomers. L-P2A-R (V109K) and L-T2A-R (V109K) indicate the P2A- and T2A-coupled ZF-ND1 monomers, respectively. The total amount of transfected plasmid DNA: L+R (V109K), 2 μg (2); L+R (V109K), 1 μg (1); L-P2A-R (V109K), 1 μg; L-T2A-R (V109K), 1 μg.
